# Behavioral and Neuroanatomical Account of Impulsivity in Parkinson's Disease

**DOI:** 10.3389/fneur.2019.01338

**Published:** 2020-01-10

**Authors:** Pavlína Hlavatá, Pavla Linhartová, Rastislav Šumec, Pavel Filip, Miroslav Světlák, Marek Baláž, Tomáš Kašpárek, Martin Bareš

**Affiliations:** ^1^Department of Psychiatry, Faculty of Medicine, Masaryk University Brno and University Hospital, Brno, Czechia; ^2^Behavioral and Social Neuroscience Research Group, CEITEC—Central European Institute of Technology, Masaryk University, Brno, Czechia; ^3^First Department of Neurology, Faculty of Medicine, Masaryk University and St. Anne's University Hospital, Brno, Czechia; ^4^Faculty of Medicine, Institute of Psychology and Psychosomatics, Masaryk University Brno and University Hospital, Brno, Czechia; ^5^Department of Neurology, School of Medicine, University of Minnesota, Minneapolis, MN, United States

**Keywords:** impulse control disorder, Parkinson's disease, impulsive action, impulsive choice, structural MRI, Iowa gambling task, delay discounting task, stop signal task

## Abstract

Impulse control disorder (ICD) is a major non-motor complication of Parkinson's disease (PD) with often devastating consequences for patients' quality of life. In this study, we aimed to characterize the phenotype of impulsivity in PD and its neuroanatomical correlates.

**Methods:** Thirty-seven PD patients (15 patients with ICD, 22 patients without ICD) and 36 healthy controls underwent a neuropsychological battery. The test battery consisted of anxiety and depression scales, self-report measures of impulsivity (Barratt scale and UPPS-P), behavioral measures of impulsive action (Go/No-Go task, Stop signal task) and impulsive choice (Delay discounting, Iowa gambling task), and measures of cognitive abilities (working memory, attention, executive function). Patients and controls underwent structural MRI scanning.

**Results:** Patients with ICD had significantly higher levels of self-reported impulsivity (Barratt scale and Lack of perseverance from UPPS-P) in comparison with healthy controls and non-impulsive PD patients, but they performed similarly in behavioral tasks, except for the Iowa gambling task. In this task, patients with ICD made significantly less risky decisions than patients without ICD and healthy controls. Patients without ICD did not differ from healthy controls in self-reported impulsivity or behavioral measurements. Both patient groups were more anxious and depressive than healthy controls. MRI scanning revealed structural differences in cortical areas related to impulse control in both patient groups. Patients without ICD had lower volumes and cortical thickness of bilateral inferior frontal gyrus. Patients with ICD had higher volumes of right caudal anterior cingulate and rostral middle frontal cortex.

**Conclusions:** Despite the presence of ICD as confirmed by both clinical follow-up and self-reported impulsivity scales and supported by structural differences in various neural nodes related to inhibitory control and reward processing, patients with ICD performed no worse than healthy controls in various behavioral tasks previously hypothesized as robust impulsivity measures. These results call for caution against impetuous interpretation of behavioral tests, since various factors may and will influence the ultimate outcomes, be it the lack of sensitivity in specific, limited ICD subtypes, excessive caution of ICD patients during testing due to previous negative experience rendering simplistic tasks insufficient, or other, as of now unknown aspects, calling for further research.

## Introduction

Parkinson's disease (PD) is a neurodegenerative disease characterized not only by motor symptoms as rigidity, hypokinesis, and tremor, but also by a variety of non-motor deficits. Among these non-motor symptoms, impulse control disorder (ICD) is the one that has a devastating effect on the quality of patients' life. In the population of PD patients, ICD manifests itself in a wide spectrum of impulsive behaviors such as pathological gambling, binge eating, hypersexuality, excessive shopping, and also repetitive excessive behaviors like punding (stereotyped purposeless repetitive behavior), hobbyism (internet use, reading, art work), walk-abouts (purposeless wandering), and hoarding (1, 2).

ICD in PD is usually believed to be a consequence of dopaminergic treatment (2, 3), but recent studies claim that there is an interaction of medication influence with underlying vulnerability to impulsive behavior (4, 5). Therefore, it is important to describe the behavioral pattern and neurobiological correlates of impulsivity in PD to track possible correlates of ICD in PD patients. Impulsivity is a heterogeneous concept, which can be understood as a personality trait or as a consequence of a neurobiological function deficit. Behavioral models of impulsivity distinguish impulsive action, which means inability to inhibit prepotent or unwanted actions (waiting impulsivity) or inability to stop ongoing action (stopping impulsivity), and impulsive choice, which includes aspects like high sensitivity to reward, risk taking, and preference of small immediate rewards to long-term gains (6). Impulsivity as a personality trait is measured by self-reported questionnaires, such as the Barratt scale or the UPPS-P scale; impulsivity as neurobiological deficit is measured by behavioral tasks.

Research using self-reported methods concluded that people with certain personality traits are in higher risk of developing ICD. Specifically, associations between ICD and higher novelty/sensation seeking, compulsivity, depression, and anxiety were found (3, 7–9). Impulsive personal traits have been previously associated with ICD in PD. Patients with ICD manifest elevated self-impulsivity in the Barratt scale (9, 10). Other risk factors for developing ICD in PD patients are younger age/younger age of disease onset, family history of behavior such as gambling or alcoholism, being man, and being single (2, 8, 11–14).

Previous research revealed increased impulsive choice, i.e., increased tendency to risky or disadvantageous decisions in general PD population (i.e., ICD status was not reported) (15–23) or in PD-ICD patients specifically (9, 24, 25). A tendency to irrational choices and early decisions in the Bead task, a test of reflection impulsivity, was observed in PD-ICD patients (26). These patients collected less information than PD patients without ICD or healthy controls before making a decision. However, results are not consistent. Study of Voon et al. (27) reported that PD-ICD patients have greater tendency to risk in comparison with patients without ICD, but only when there is only gain possibility, not in a situation with possibility of loss. Several studies did not find any differences in choice impulsivity between PD-ICD patients and healthy controls or PD patients without ICD (28–30) or in general PD population (31, 32). One study reported only statistical trend toward more risky decisions in the PD-ICD group in the Iowa gambling task (10). No significant differences in the performance between patients with and without ICD were reported in the Balloon Analog Risk Task (33–35). Ambivalent results were obtained regarding learning from the negative feedback. Several studies found lower sensitivity to negative feedback in PD-ICD (33, 36, 37), but others found no differences between ICD and patients without ICD or healthy controls (11, 35). Moreover, PD-ICD patients without medication showed decreased learning from negative feedback and increased learning from positive feedback compared to the PD-ICD patients on medication (11). Patients with ICD on dopamine agonists make faster decisions and more impulsive choices than patients off medication (28) and show enhanced sensitivity to risk (27).

Several studies reported increased stopping impulsivity (38–41) and waiting impulsivity (42) in the general PD population. However, there are also reports of intact impulsive action in PD patients without ICD (43) and in PD patients in general (ICD not specified) (44). Studies conducted on PD-ICD patients brought evidence of intact impulsive action (10, 45, 46); one study reported even faster Stop signal reaction time (SSRT), which means better ability to stop ongoing actions, i.e., lower stopping impulsivity in PD-ICD patients (43).

Structural imaging methods found evidence of cortical thickness abnormalities in PD-ICD in the structures related to impulse control and decision making, namely, the orbitofrontal cortex (OFC), anterior cingulate cortex (ACC), dorsolateral prefrontal cortex (DLPFC), and corpus callosum (47–51). A gray-matter volume loss in amygdala and orbitofrontal cortex and volume changes in nucleus accumbens (NAcc) and in amygdala were observed in PD-ICD (21, 47, 48, 52).

There are only few studies directly comparing PD patients with and without ICD in impulsivity domains. Moreover, individual studies usually do not target multiple impulsive domains simultaneously, despite the heterogeneous nature of impulsivity. The aim of our study was to describe the phenotype of impulsivity in PD patients with and without ICD by testing multiple impulsivity domains. We included three groups into our research: PD patients with ICD (PD-ICD), PD patients without ICD (PD-nonICD) and healthy controls (HC). We used self-reported impulsivity questionnaires (the UPPS-P and the Barratt scale), behavioral tasks for assessment of impulsive action (Go/No-Go task, Stop signal task), and impulsive choice (Iowa gambling task, Delay discounting task). We also measured depression, anxiety, and cognitive components influencing performance in impulsivity tasks (attention, working memory, and executive functioning). Moreover, structural magnetic resonance images (MRI) were obtained. We analyzed several brain regions, which were previously in the literature linked to impulsivity, inhibitory control, and decision making (**Table 3**).

Based on previous research, we hypothesized that PD patients with ICD, but not those without ICD, show greater impulsivity in self-reported scales. Further, we hypothesized that only PD-ICD patients show increased impulsivity in the Sensation seeking subscale of UPPS-P in comparison with healthy controls. We did not make any specific hypotheses for the other UPPS-P dimensions due to the lack of literature. We expect greater impulsive choice in the PD-ICD population, but intact impulsive action. No specific hypotheses were made about impulsive action and impulsive choice in patients without ICD due to conflicting literature and lack of literature targeting specifically PD patients without ICD. We expected PD-ICD patients to show brain structure abnormalities in comparison with healthy controls in the prefrontal cortex (orbitofrontal cortex, ACC, and DLPFC) and NAcc.

## Methods

### Subjects

Thirty-seven patients with Parkinson‘s disease and 36 healthy controls took part in this study; the groups were matched by age, gender, education, and laterality. All patients were recruited from the University Hospital of St. Anne, Brno, Czech Republic. Healthy volunteers were recruited by advertising in local newspapers. Patients aged 25–75 with a diagnosis of Parkinson‘s disease based on the UK Brain Bank Criteria (53) were recruited in the study. All patients were under dopaminergic medication for at least 12 months preceding the examination and had stable doses at least for 4 weeks before testing. Exclusion criteria for all subjects were neurological or systemic disorder with effect on brain function (except PD in the patient groups), lesions in MRI scans, comorbid psychotic disease, affective disease or autism spectrum disorder, mental retardation, cognitive deficit (MMSE under 27), severe depression, and substance abuse.

Prior to the testing, data about age of the PD onset, Hoehn and Yahr stage, and medication calculated as levodopa equivalent dose were obtained, and cognition was examined using MMSE. Healthy volunteers were assessed by the Mini international neuropsychiatric interview [MINI; (54)] to exclude any subjects with psychiatric symptoms. The patients had no self-reported cognitive problems; none of them scored below 28 in Mini-Mental State Examination [MMSE; (55)]. Most of the patients were in stage 2 or 3 according to the Hoehn and Yahr Scale (56). Fifteen patients had ICD, and 22 patients were without ICD. Patients with ICD were selected by a neurologist based on interview with the patient and presence of ICD signs in their medical records. Only patients with severe behavioral problems connected to ICD were recruited. The patients showed the following ICD symptoms: gambling (5 patients), binge eating (3 patients), hypersexuality (2 patients), hobbyism (1 patient), compulsive buying (1 patient), hoarding (1 patient), punding (1 patient), pedantry (1 patient), and excessive cleaning (1 patient). Some of the patients showed more than one symptom of ICD. The study was approved by the Institutional Ethical Committee. All participants signed the informed consent prior to the beginning of the procedure. Characteristics of the sample are summarized in Table 1.

**Table 1:** Demographic, neurologic data and neuropsychological data of PD, PD-ICD and HC groups.

	**PD-ICD (*n* = 15)**	**PD-nonICD (*n* = 22)**	**Healthy controls (*n* = 36)**	**Statistics value, *p***	**Effect size**
Gender M/F	11/4	10/12	19/17		
Age	59.27 (8.88)	69.18 (5.47)	66.14 (7.70)	*F*_(2, 70)_ = 8.214, *p* = 0.001	
Education	6.7, 13.3, 46.7, 33.4	4.5, 13.6, 68.2, 13.6	0, 16.7, 55.6, 27.7	$\chi^{2}(2)$ = 0.658, *p* = 0.658	
Socioeconomic status	6.7, 33.3, 46.7, 6.7	13.6, 22.7, 54.5, 9.0	2.8, 19.4, 52.8, 22.2	$\chi^{2}(2)$ = 3.902, *p* = 0.142	
**Neurologic data**					**d**
H a Y stage	2.53 (0.64)	2.48 (0.66)		*p* = 0.799, *t* = 0.256	*d* = 0.085927
Age of onset	50.80 (9.64)	62.55 (6.25)		*p* < 0.001, *t* = −4.160	*d* = 1.445757
Diseases duration	8.87 (4.17)	6.95 (4.63)		*p* = 0.208, *t* = 1.282	*d* = 0.433676
L-dopa equivalent dose	1289.75 (543.97)	1025.46 (567.18)		*p* = 0.166, *t* = 1.414	*d* = 0.475602
**Neuropsychological data**					**η**^**2**^
MADRS	3.73 (5.68)	3.05 (3.84)	0.69 (1.83)	*F*_(2, 70)_ = 5.202, *p* = 0.008	η^2^ = 0.129
SAS score	36.73 (5.40)	35.73 (5.25)	28.50 (6.59)	*F*_(2, 70)_ = 14.980, *p* < 0.001	η^2^ = 0.300

*Education (%), Primary, lower secondary, higher secondary, college*.

*Socioeconomic status (%), insufficient, unsatisfactory, satisfactory, very satisfactory*.

*MADRS, Montgomery Asberg Depression Rating Scale; SAS, Zung Self-reported Anxiety Scale; M, male; F, female*.

### Experimental Procedure

Subjects underwent a neuropsychological battery of tests and questionnaires and performed four computerized behavioral tasks. The test battery included the Barratt scale (57) and the UPPS-P scale (58–60) for measuring self-reported impulsivity, the Montgomery-Asberg Depression Rating Scale (61), the Zung Self-Rating Anxiety Scale (62), and standardized measures of cognitive functions. Waiting impulsivity was measured by the Go/No-Go task (GNG), stopping impulsivity was measured by the Stop signal task (SST), and impulsive choice was assessed by the Delay discounting task (DDT) and the Iowa gambling task (IGT). Results are summarized in Table 2. Behavioral tests were created in E-Prime 2.0 software. The whole testing procedure took ~2.5 h.

**Table 2:** Descriptive statistics of dependent variables and results of ANOVAs comparing PD-nonICD, PD-ICD, and HC groups.

	**PD-ICD (*n* = 15)**	**PD-nonICD (*n* = 22)**	**Healthy controls (*n* = 36)**	**Statistic value, *p***	**Effect size**
Barrat scale	60.47 (7.54)	54.14 (5.60)	54.31 (6.48)	*F*_(2, 70)_ = 5.537, *p* = 0.006	η^2^ = 0.137
UPPS-P PRE	20.60 (5.05)	19.77 (4.36)	18.67 (4.03)	*F*_(2, 70)_ = 1.162, *p* = 0.319	η^2^ = 0.032
UPPS-P PER	20.33 (5.08)	19.14 (3.58)	16.83 (3.48)	*F*_(2, 70)_ = 5.140, *p* = 0.008	η^2^ = 0.128
UPPS-P SS	22.07 (6.78)	23.64 (6.76)	26.25 (6.85)	*F*_(2, 70)_ = 2.317, *p* = 0.106	η^2^ = 0.062
UPPS-P NU	27.27 (6.44)	25.18 (6.54)	25.51 (6.22)	*F*_(2, 69)_ = 0.537, *p* = 0.587	η^2^ = 0.015
UPPS-P PU	28.33 (7.51)	28.27 (6.99)	26.69 (8.25)	*F*_(2, 70)_ = 0.391, *p* = 0.678	η^2^ = 0.011
Go omissions %	18.95 (5.57)	18.76 (7,80)	16.94 (5.96)	*F*_(2, 70)_ = 0.782, *p* = 0.461	η^2^ = 0.022
Go RT	419.36 (50.98)	418.03 (48.11)	402.83 (56.48)	*F*_(2, 70)_ = 0.808, *p* = 0.450	η^2^ = 0.023
No-Go commissions %	18.65 (12.36)	25.97 (19.51)	22.45 (17.21)	*F*_(2, 70)_ = 0.827, *p* = 0.442	η^2^ = 0.023
SSRT	298.46 (125.45)	242.36 (115.86)	281.97 (80.90)	*F*_(2, 69)_ = 1.580, *p* = 0.213	η^2^ = 0.044
DD k 990	0.022 (0.046)	0.061 (0.125)	0.019 (0.050)	*F*_(2, 65)_ = 1.886, *p* = 0.160	η^2^ = 0.026
DD k 24900	0.005 (0.0103)	0.077 (0.385)	0.004 (0.005)	*F*_(2, 69)_ = 0.626, *p* = 0.538	η^2^ = 0.018
DD AUC 990	0.61 (0.31)	0.43 (0.29)	0.58 (0.34)	*F*_(2, 68)_ = 1.854, *p* = 0.164	η^2^ = 0.052
DD AUC 24900	0.76 (0.25)	0.71 (0.23)	0.76 (0.30)	*F*_(2, 68)_ = 0.251, *p* = 0.779	η^2^ = 0.007
IGT NET score 1st part	13.80 (29.33)	3.18 (25.80)	7.17 (25.28)	*F*_(2, 70)_ = 0.729, *p* = 0.486	η^2^ = 0.020
IGT NET score 2nd part	39.07 (44.89)	−0.91 (33.84)	15.00 (37.43)	*F*_(2, 70)_ = 4.925, *p* = 0.010	η^2^ = 0.123
IGT B % 1st part	24.55 (9.03)	32.55 (10.72)	31.00 (11.27)	*F*_(2, 70)_ = 2.723, *p* = 0.073	η^2^ = 0.072
IGT B % 2nd part	16.86 (13.74)	36.64 (17.04)	30.58 (15.98)	*F*_(2, 69)_ = 6.696, *p* = 0.002	η^2^ = 0.163
Digit span	14.47 (2.72)	14.18 (3.00)	14.67 (3.87)	*F*_(2, 68)_ = 0.138, *p* = 0.872	η^2^ = 0.004
d2 speed	119.07 (24.99)	106.80 (24.50)	126.06 (19.99)	*F*_(2, 66)_ = 4.624, *p* = 0.013	η^2^ = 0.123
d2 accuracy (error %)	9.25 (8.78)	8.67 (6.06)	9.39 (6.21)	*F*_(2, 66)_ = 0.074, *p* = 0.929	η^2^ = 0.002
ToL moves	34.79 (17.08)	40.90 (24.29)	31.08 (19.67)	*F*_(2, 68)_ = 1.493, *p* = 0.232	η^2^ = 0.042
ToL init. time	137.21 (71.74)	91.25 (42.32)	109.81 (55.98)	*F*_(2, 68)_ = 2.841, *p* = 0.065	η^2^ = 0.077
ToL exec. time	337.71 (164.86)	360.10 (175.52)	278.33 (141.59)	*F*_(2, 68)_ = 2.000, *p* = 0.143	η^2^ = 0.056

*PRE, Lack of premeditation; PER, Lack of perseverance; SS, Sensation seeking; NU, Negative urgency; PU, positive urgency; Go RT, Go reaction time; SSRT, Stop signal reaction time; DD, delay discounting; AUC, Area under the curve; IGT, Iowa gambling task; IGT B %, percentage of B deck cards selections; ToL, Tower of London; moves, total number of moves made during the task; ToL inic. time; ToL exec. time*.

### Impulsivity Questionnaires

We used the validated Czech translation of the Barratt scale for assessing impulsivity (57). This method includes aspects of non-planning impulsiveness, attentional impulsiveness, and motor impulsiveness. However, the three-factor structure has been questioned [e.g., (63, 64)]; therefore, only total score for this scale was calculated. Self-reported impulsivity was also measured by the Czech validated translation of the UPPS-P scale (58–60). The questionnaire consists of five subscales: Lack of premeditation (11 items), Lack of perseverance (10 items), Sensation seeking (12 items), Negative urgency (12 items), and Positive urgency (14 items), where urgency indicates the tendency to act impulsively under influence of emotions.

### Behavioral Tasks

#### GNG Task

The GNG task contained two types of stimuli, the letter *A* was a Go stimulus and the letter *B* was a No-Go stimulus. Stimuli were in white color presented on the black screen. Subjects had to quickly react by pressing a key when letter *A* appeared and avoid the reaction when letter B appeared. A fixation cross preceded each stimulus. The whole task consisted of four blocks with 48 trials in each block. All subjects performed a short practice before the testing. The stimulus duration was 0.4 s; fixation cross duration varied from 1.1 to 2.6 s. Three parameters were analyzed from this task: No-Go commission errors percentage (percentage of erroneous key press after No-Go trial), Go omission errors percentage (percentage of Go trials erroneously followed by no key press), and Go reaction time (average reaction time on correct Go trials). Go stimuli occurred in 83% and No-Go stimuli occurred in 17%; this ratio makes the task more cognitively demanding and the subjects prone to make more commission errors.

#### SST

Stimuli in the SST were white arrows pointing to the left or to the right; subjects had to press an arrow key pointing to the same direction as the arrow presented on the black screen. However, the subjects had to stop their reaction and press no key when the stimulus was followed by a visual stop signal (the arrow turned red). Stop signals appeared in 25% of trials. Delays in the presentation of stop signal (stop signal delay, SSD) varied during the task in order to prevent the subject from developing response pattern, the starting latency was 200 ms. When the subject succeeded in the trial, the latency increased by 45 ms; when the subject failed, the latency decreased by 45 ms. Each stimulus lasted until the subject reacted by pressing a key or for 1 s if there was no reaction from the subject. A fixation cross appeared before every stimulus; the duration of the cross was again variable between 1.1 and 2.6 s. The task consisted of four blocks with 48 trials in each block; a short practice preceded the testing procedure. The SSRT was calculated by subtracting the average SSD from the average Go reaction time. SSRT refers to the time that the subject needs to stop his/her reaction; the longer time needed, the more difficult it is to interrupt one's own actions; i.e., higher SSRT is linked with higher impulsivity.

#### DD

In the DD task, subjects were asked to choose between two possible rewards in every trial—a smaller but immediate reward or higher but delayed reward. For example, *Would you prefer to receive 510 CZK today or 990 CZK in a week?* There were five possible delays—1 day, 1 week, 1 month, 3 months, and 6 months—and two delayed reward levels 990 CZK (around 40 EUR) and 24900 CZK (around 980 EUR). These delays and rewards were set according to the pilot study. Questions with different combinations of delays and delayed rewards were presented in random order and the subject was asked if he or she prefers the immediate or the delayed reward. The amount of immediate reward varied in intervals of 20 CZK (in case of smaller delayed reward) or 500 CZK (in case of bigger delayed reward) until an indifference point, where the subjective value of the immediate reward is equal to the subjective value of the delayed reward, was found.

Two parameters were calculated for each delayed reward (*k* parameter and area under the curve). The hyperbolic discount parameter (*k*) was calculated by the equation DR = I/(k^*^D), where DR is delayed reward, I is immediate reward, and D is delay. Naturally, as the reward delay increases, the subjective value of this reward decreases (65). Larger values of *k* indicate steeper decline of the subjective value and therefore greater impulsivity. The second parameter was the area under the curve; the curve is estimated by the connection of indifference points for each delay reward. The lower the AUC means the steeper discounting and the higher impulsive choice (66).

#### IGT

The computerized version of IGT consisted of 200 trials (67, 68). In every trial, subjects had to make a series of choices between four card decks by pressing the key with number of the chosen deck. Two of the decks are disadvantageous (A and B) and two decks are advantageous (C and D). Subjects were informed that some of the decks were better than the other and they were instructed to play until the game ends and try to win as much money as possible. However, participants didn't know which decks are bad or good, how many trials had the game, or risk of losses in each deck. Impulsive behavior is associated with decks A and B. Deck A contains high immediate rewards with high risk of loss, and deck B contains high immediate rewards with low risk of very high loss. IGT net score was calculated by subtraction of disadvantageous deck choices from advantageous deck choices [(C + D) − (A + B)]. We also compared the relative frequency of A and B deck choices.

### Cognitive Abilities and Executive Functions

For working memory, the total score of Digit span subtest from WAIS-III (69) was analyzed. Three parameters from Tower of London (70) were calculated for assessment of executive functions—total move score, total initiation time, and total execution time. Attention was measured by test d2-R (71) with two analyzed parameters—speed (total number of processed items) and accuracy (percentage of omission and commission errors).

### MRI Data Acquisition and Analysis

After the behavioral testing, the patients and controls underwent MRI scanning. Ten patients and eight controls did not undergo the acquisition due to MRI contraindications or inability to tolerate the procedure. The scanning was performed using a 3-T MRI scanner, SIEMENS MAGNETOM Prisma syngo (Siemens Medical Systems, Erlangen, Germany) at the Central European Institute of Technology of Masaryk University, Brno, Czech Republic. A high-resolution T1-weighted scan was acquired using the following parameters: MPRAGE sequence with repetition time = 2,300 ms, echo time = 2.34 ms, flip angle = 8°, voxel size 1.00 × 1.00 × 1.00 mm, matrix 240 × 224 × 224. Further three patients were excluded from the scanning because of excessive head movement resulting to significant motion artifacts not compatible with the automatic processing pipeline. The final size of the sample that competed MRI session was 16 PD-nonICD patients, 8 PD-ICD patients, and 28 healthy controls.

Volumetric segmentation was performed in this T1-weighted scan using the standard automated pipeline (72) implemented in the FreeSurfer image analysis suite, version 5.3.0 (http://surfer.nmr.mgh.harvard.edu/). The accuracy of the segmentation in each subject was visually inspected by a trained operator (P.F.). The volumes (adjusted for the estimated intracranial volume) and surface areas (in case of cortical regions) of several regions of interest (Table 3) and the volume of the whole brain, as provided by the automatic segmentation algorithm of FreeSurfer, were compared between the PD-nonICD, PD-ICD patients and healthy controls using one-way analysis of variance (ANOVA) with Bonferroni *post-hoc* multiple-comparison correction.

**Table 3:** Anatomical regions of interest.

**Anatomical region**	**Side**
Caudate	R,L
Putamen	R,L
Nucleus accumbens	R,L
Accumbens area	R,L
Caudal anterior cingulate	R,L
Rostral anterior cingulate	R,L
Caudal middle frontal	R,L
Rostral middle frontal	R,L
Lateral orbitofrontal	R,L
Medial orbitofrontal	R,L
Pars orbitalis	R,L
Pars triangularis	R,L
Precentral gyrus	R,L
Insula	R,L

*L, left; R, right*.

We performed statistical comparisons (two-sample *t*-tests) of subgroup of patients who underwent MRI scanning with the subgroup of patients who did not undergo MRI scanning to be sure that the MRI subgroup was not significantly different from the original patient group in the behavioral, demographic, and other measured parameters.

### Statistical Analysis

Data were analyzed in IBM SPSS Statistics 24 software. We compared demographic characteristics, performance in computerized tasks, cognitive tasks, scores from questionnaires, and MRI parameters between groups of healthy controls, Parkinson patients with ICD, and Parkinson patients without ICD. The data from computerized behavioral tasks, questionnaires, and cognitive tasks were compared by one-way ANOVA without covariates. In cases of significant group differences, Tukey's *post-hoc* tests were applied. Sociodemographic characteristics (education, socioeconomic status) were analyzed by Kruskal–Wallis *H*-test; neurological data of the two patient groups were analyzed by independent *t*-tests. IGT net scores of the first and the second part of the task were analyzed in *jamovi* software by repeated measures ANOVA with Tukey's *post-hoc* tests with time (first vs. second part of the task) as within-subject factor and group as between-subject factor. Results are reported at *p* < 0.05 level of significance.

## Results

### Demographic Data

The three groups did not differ in education or socioeconomic status (Table 1). The groups were also matched by age; the healthy controls were selected to have similar age to patients with 2 years tolerance. However, there was a difference in age between groups; patients with ICD were significantly younger than those without ICD and healthy controls (PD-nonICD vs. PD-ICD: *p* < 0.001, PD-ICD vs. HC: *p* = 0.009). The PD-ICD patients were younger than PD-nonICD patients at the time of the disease onset (PD-ICD vs. PD-nonICD: *p* < 0.001). There were no differences in disease duration, levodopa equivalent dose, or Hoehn and Yahr stage between clinical groups.

The group of patients who underwent MRI scanning did not differ from the group of patients without MRI in the cognitive abilities, behavioral parameters, or self-reported impulsivity. However, the groups significantly differed in age (*p* = 0.028, group with MRI mean = 67.56, *SD* = 6.4; group without MRI mean = 58.7, *SD* = 10.5).

### Depression and Anxiety

There was a significant effect of group on scores of anxiety and depression. Both clinical groups (PD-ICD and PD-nonICD) had higher scores of depression than healthy controls (MADRS score PD-ICD vs. HC: *p* = 0.018, PD-nonICD vs. HC: *p* = 0.043). Both clinical groups also had significantly higher levels of anxiety than healthy controls (SAS score PD-ICD vs. HC: *p* < 0.001, PD-nonICD vs. HC: *p* < 0.001). No significant differences were detected between PD-nonICD and PD-ICD patients in anxiety or depression.

### Impulsivity Questionnaires

There were significant group differences in the Barratt scale score. The PD-ICD group scored the highest on the Barratt scale, which means that PD-ICD patients were more impulsive than PD-nonICD patients (*p* = 0.013) and controls (*p* = 0.008). The PD-nonICD group and control group did not differ in the Barratt scale score (*p* = 0.995). In the UPPS-P scale, patients with ICD showed elevated score, as compared to healthy controls, in Lack of perseverance (*p* = 0.017). No differences between the groups were found in the other UPPS-P subscales. The PD-nonICD group did not differ from healthy controls in any of the self-reported impulsivity measurements and did not differ from PD-ICD except for the Barratt scale score.

### Behavioral Tasks

There was no significant effect of group on performance in GNG, DDT, or SST (Table 2). Both groups of patients had similar RTs, accuracy, SSRT, and discounting parameters as healthy controls. Group differences were found in performance in IGT. Analysis revealed significant effects of time, group, and time vs. group interaction on the IGT net score [(C + D) − (A + B)]. There were no significant differences in the NET score in the first part of the task, but the PD-ICD group had significantly higher NET score than the PD-nonICD group (*p* = 0.007) in the second part of the task. No significant differences were found between healthy controls and PD-nonICD or between healthy controls and PD-ICD. There was significant effect of time vs. group interaction [*F*_(2, 70)_ = 3.36, *p* = 0.041]. *Post-hoc* tests revealed that only the PD-ICD group improved their NET score in the second half of the task; the difference was marginally significant in PD-ICD (*p* = 0.055) and not significant in other groups (HC: *p* = 0.733, PD-nonICD: *p* = 0.993). We further made analysis of particular deck selections in the first and second half of the task. There was a group difference in B deck preference in the second half of the task; specifically, the PD-ICD group selected cards from the B deck less likely than the PD-nonICD group (PD-ICD vs. PD-nonICD: *p* = 0.002) and healthy controls (PD-ICD vs. HC: *p* = 0.021). No significant differences were found in A deck selection relative frequency.

### Cognitive Abilities and Executive Functions

The three groups did not differ in working memory (Digit span). ANOVA revealed significant group differences in processing speed in d2 test. Patients without ICD were significantly slower than healthy controls (PD-nonICD vs. HC: *p* = 0.009). No significant differences were found between the PD-ICD patients and healthy controls or between the two patients' groups in the accuracy in d2 test. No significant differences were detected in total move score and total execution time in the Tower of London. However, we observed a trend in total initiation time between the PD-nonICD and PD-ICD group. The PD-ICD group showed longer incitation time than PD-nonICD group, but the comparison was only marginally significant (PD-nonICD vs. PD-ICD *p* = 0.052). All groups worked with similar accuracy in the test.

### MRI

PD-nonICD patients differed from HC in the estimated intracranial volume (eICV) of the right nucleus accumbens area (*p* = 0.002), bilateral pars orbitalis (*p* < 0.001), the left pars triangularis (*p* = 0.002), and the left precentral gyrus (*p* = 0.032) (Table 4); PD-nonICD patients showed decrease of volumes in all of these regions (All presented *p*-values are Bonferroni-corrected for multiple comparisons.) PD-nonICD patients also showed cortical thinning of the left pars orbitalis (*p* = 0.020) in comparison with HC. PD-ICD patients in comparison with HC showed increase of the right caudal anterior cingulate area (*p* = 0.003) and right rostral middle frontal area (*p* = 0.018) and also increase of total volumes of these regions (right caudal ACC, *p* = 0.010, right rostral middle frontal *p* = 0.007). However, differences in eICV volumes in these areas did not reach significance in the case of PD-ICD group. Both groups—PD-nonICD and PD-ICD—showed a decrease in the eICV volume of the right precentral gyrus in comparison with HC, but none of the results reached significance in *post-hoc* testing (*p* > 0.05). In the comparison of PD-nonICD and PD-ICD, only total volumes of bilateral pars orbitalis (right *p* = 0.029, left *p* = 0.039) and right caudal anterior cingulate (*p* = 0.041) differed, with PD-ICD patients having greater volumes. Comparison of cortical areas, eICV volumes, or cortical thickness between the two patient groups did not bring any significant results.

**Table 4:** Results of anatomical structures comparisons—significant contrasts.

**Volumes**	***p***	***F***	**Significant contrasts**
Right accumbens area	0.022	4.130	PD-nonICDxHC
lh pars orbitalis	0.013	4.737	PD-nonICDxHC, PD-nonICDXPD-ICD
rh caudal anterior cingulate	0.012	4.850	PD-nonICDXPD-ICD, PD-ICDxHC
rh pars orbitalis	0.005	5.841	PD-nonICDXPD-ICD, PD-nonICDxHC
rh rostral middle frontal	0.009	5.202	PD-ICDxHC
**Volumes eICV**			
Right accumbens area	0.003	6.688	PD-nonICDxHC
lh pars orbitalis	0.000	10.515	PD-nonICDxHC
lh pars triangularis	0.002	7.074	PD-nonICDxHC
lh precentral gyrus	0.013	4.718	PD-nonICDxHC
rh pars orbitalis	0.000	13.144	PD-nonICDxHC
rh precentral gyrus	0.021	4.160	No significant contrast
**Thickness**			
lh pars orbitalis	0.021	4.171	PD-nonICDxHC
**Area**			
rh caudal anterior cingulate	0.005	6.006	PD-ICDxHC
rh rostral middle frontal	0.022	4.146	PD-ICDxHC

*Volumes eICV, estimated intracranial volume; lh, left hemisphere; rh, right hemisphere*.

## Discussion

In the study, several possible factors associated with ICD were compared across the groups (anxiety, depression, age, age of onset, disease stage, and duration). Among these factors, only age of onset and age differed between the ICD and non-ICD group. Patients with ICD were younger and had lower age of disease onset than patients without ICD. Our results support previous research reporting association between PD-ICD and younger disease onset or younger age (2, 11–14).

Elevated self-reported impulsivity in the Barratt scale in PD-ICD in comparison with HC and PD-nonICD is consistent with previous research in this population (9, 10, 13, 73). In the UPPS-P scale, our results did not confirm our hypothesis regarding elevated Sensation seeking in PD-ICD. The absence of differences in Sensation seeking in PD-ICD might be surprising, when some other studies found associations of higher Sensation/Novelty seeking with ICD in this population (7–9, 13) [but see Evans et al. (12)]. The differences might be due to using various measurement tools with different concepts of Sensation seeking. Regarding the UPPS-P dimensions, we found only one study that targeted these dimensions in PD-ICD (7). This work reported increased impulsivity in Lack of premeditation, Urgency, and Sensation seeking in PD-ICD in contrast with HC and elevated Sensation seeking in contrast with PD-nonICD patients. However, only the shortened 16-item UPPS scale was used and the scale was answered by close relatives of the patients not the patients themselves, which may cause inconsistency of their results with our findings. PD-ICD patients in contrast with HC showed elevated score only in the Lack of perseverance. Perseverance is defined as “the ability to remain focused on a task that may be boring and/or difficult.” Lack of perseverance has been associated with poor resistance to distraction, harm avoidance, poor concentration on boring or difficult tasks, lower responsibility perception, and difficulties in dealing with frustration (74). Lower perseverance was reported in people with obsessive–compulsive symptoms, compulsive buying (74–77), bulimia (78), and self-injury behavior (79). One study found lower perseverance in PD patients with and without ICD (7). PD-ICD patients in our sample did not manifest higher levels of Urgency, which represents the aspect of emotional impulsivity. Taken together with no impairments in impulsive choice, it seems that impulsivity in PD-ICD is not elevated by strong emotions unlike, for example, borderline disorder, where impulsivity is more prominent in intense emotional state (6, 80, 81). PD-ICD patients are more likely to avoid harm and lower their effort in boring or difficult situations. They have impaired ability to maintain long-term goal and facing the obstacles; when facing difficulties, they rather abandon the goal. Even though UPPS-P questionnaire is the most up-to-date personality model of impulsivity, it is not used in studies conducted on PD-ICD populations. Future research with larger sample sizes studying PD-ICD in the context of UPPS-P impulsivity model would be beneficial.

In accordance with our hypothesis, PD-ICD patients in our study did not show impairments in impulsive action (waiting and stopping impulsivity). Results were also negative for the non-impulsive group. Previous studies of impulsive action in general PD population brought mixed results (38–44). There might be several factors responsive for the inconsistency in impulsive action among studies. Differences in results can be caused by cognitive impairments in patients' groups, heterogeneity in the patients' samples, and by differences in task designs. Patients in studies have different clinical characteristics such as different disease stage and duration, cognitive impairments, and different severity of anxiety and depression. Some of the studies included patients with cognitive impairments and elevated depression in their samples (38, 45). Presence of cognitive deficits and depression were linked to lower efficacy of inhibitory performance in impulsive action tasks (38, 82). Further, patients with older age and later disease stages have greater difficulties when inhibiting their responses (38). Thus, it is possible that increased impulsive action observed in some studies in the previous literature might have been influenced more by impaired cognitive functioning or psychomotor slowing associated with markedly increased depression, rather than with increased impulsivity itself.

We expected elevated choice impulsivity in PD-ICD in comparison with healthy controls, but our results did not support this expectation. None of the clinical groups differed from healthy controls on Delay discounting. Unimpaired performance of PD-ICD patients on Delay discounting is in contrast with two studies (9, 24), which found elevated discounting in PD patients with ICD in comparison with HC and non-impulsive PD patients. However, PD-ICD patients in these studies had either lower IQ or working memory deficits or were more depressed than non-impulsive patients. All of these factors were previously associated with greater discounting (83–86). Consistently with our findings, another study (28) reported similar delay discounting comparing PD-ICD and HC. The results of DD studies in PD-ICD population remain inconsistent. It is again possible that presence of cognitive impairments or elevated depression could influence performance in this task in previous studies. Future research should focus on relationship of depression, cognitive deficits, and behavioral measures of impulsivity in this population.

In the IGT in our study, patients with ICD performed surprisingly the best from all groups. PD-ICD as the only group improved their performance in the second part of the task and made significantly less risky choices. PD-ICD patients differed from PD-nonICD and HC in B deck card preference. B deck is a deck with low frequent but very high losses and high frequent gains. This deck is disadvantageous in long-term outcome. Therefore, according to the basic IGT assumption, the frequency of B deck choice declines during the task in a healthy population (67). This basic IGT assumption is being questioned, since the high preference of B deck choices observed in a healthy population in many studies suggests that it is rather the gain–loss frequency than the long-term outcome that influences decision-making in IGT in a healthy population (87). Our results suggest that PD-ICD patients, unlike HC and patients without ICD, are less sensitive to gain-loss frequency and more sensitive to high punishments. Previous studies brought evidence of impaired IGT performance in PD patients (ICD not specified) (16–23, 88) and in pathological gamblers with Parkinson (25). The difference between our study and that of Rossi et al. (25) is that we also included other forms of ICD beside pathological gambling. We recruited some patients with gambling history into our sample. It is possible that these patients with gambling history were more aware of potential risk because of their previous problems and they already developed some strategies, which helped them during the task. In contrast with previous studies, we used a prolonged protocol consisting of 200 trials. The differences between groups were more prominent in the second part of the tasks where PD-ICD patients chose the cards from the disadvantageous deck less frequently than in the first part. It seems that this prolonged version is more sensitive to describe learning from the consequences of the traditional 100 card version. We further analyzed not only the NET score but also the preference of particular packages. This four-deck approach seems to be useful, because it provides more information about decision strategies of participants.

Having in mind the age differences between groups of patients in our study, the role of age in the context of IGT results should be considered. PD-ICD in our study performed better than PD-nonICD, and they were also younger than PD-nonICD patients. Biars et al. (29) reported that increasing age negatively influenced IGT performance. Some studies found older adults to have greater tendency to less advantageous decisions in IGT than younger adults (89, 90). It is possible that older adults use different cognitive strategies and are not able to learn from the feedback as effectively as younger adults (89). On the other hand, studies reporting impaired performance in the PD or PD-ICD group in IGT found no significant age differences between compared groups (16, 18, 20–22, 25). Further investigation in this field would be beneficial.

As for cognitive functions, patients did not show any significant impairments except slower processing speed in the d2 test in PD non-ICD in contrast with HC. It may be surprising, because a decline of cognitive functions is linked to PD and cognitive impairment was previously find in patients with ICD (5). However, only patients with high MMSE scores were recruited, and therefore, those with potential cognitive problems were excluded from the testing. The slower processing speed of PD-nonICD patients in contrast to HC in d2 can be attributed to age, because the PD-nonICD group was the oldest one. Slower processing speed can also indicate attentional problems. It is possible that PD-nonICD patients are able to complete the task with similar accuracy as other experimental groups, but it costs them more effort; as a result, they need more time to process the same amount of items.

Although there were no impairments in PD-ICD on behavioral tasks, MRI scanning revealed structural differences between the three groups in the structures involved in inhibitory control and decision making. PD-ICD patients showed volume increase of ACC in comparison to PD patients and healthy controls. Our results correspond with previous research, which found increased thickness of ACC cortex in PD-ICD (48, 49, 51). Decreased functional connectivity of ACC with nucleus accumbens was previously associated with impulsive–compulsive behavior in PD (51). ACC is an important component of the reward system. It is involved in subjective evaluation of immediate and delayed rewards in delay discounting (91). Some researchers suggest that this structure is also important for conflict monitoring in situations with low predictability and high error rate; when the subject is required to adjust behavior after error response, it is needed for learning from the negative feedback (49, 92). Considering an increase in ACC volume together with the improvements in PD-ICD group during the second part of the IGT task, it suggests that PD-ICD patients were the most sensitive to the negative feedback. PD-ICD patients in our study showed an increase of volume and area in the right rostral middle frontal region in comparison with HC. Middle frontal region abnormalities in PD-ICD population were previously reported Biundo et al. (47) and Yoo et al. (50), who observed cortical thinning in PD-ICD. Increased activity of the right middle frontal gyrus was previously observed during impulsive action tasks (93). This region is associated with working memory, norm- or rule-related behavior, and making strategic decisions in social context (94, 95).

However, our results are not entirely consistent with previous research. One study of PD-ICD patients reported no structural differences between PD-ICD and PD-nonICD or HC (96). Recent research brought also evidence of structural differences in orbitofrontal cortex and nucleus accumbens in PD-ICD (47–49), but our group comparisons were negative regarding these regions. Differences in anatomical findings across the studies might be caused by different analysis approaches and further by the presence of cognitive impaired individuals in some of the studies, as well as by variability of ICD patients in behavioral manifestations of ICD, in disease duration and disease progression. Disease progression and symptom severity are very important factors. For example, PD-ICD patients in the study of Pellicano et al. (48), which observed more extensive structural changes than our study, were in more advanced stage and showed more severe disease symptoms than non-impulsive patients. On the other hand, the study of Ricciardi et al. (96), which did not find any structural differences, included patients in earlier stage of the disease and with shorter disease duration than our study. Some studies tested patients with mild cognitive impairment (47, 49). Most of the studies included patients with variable ICD symptoms often with more than one manifestation of ICD. We also included variable ICD sample into our study. It is possible that particular subtypes of impulsive behavior like gambling, hypersexuality, or binge eating differ from each other in behavioral impulsivity or in their neurobiological correlates.

Patients without ICD differed from healthy controls in the regions of inferior frontal gyrus, right nucleus accumbens, and left precentral gyrus. These findings also indicate that patients without ICD have some brain pathology in regions relevant for impulsivity. This corresponds with previous MRI research conducted on the general PD population (42, 97–100).

This study has several limitations. First of all, the research sample consisted of a small number of patients due to the inclusion of only patients with severe impulsive problems and avoiding patients with cognitive deficit. Even though the prevalence of ICD in the population is higher, we decided to include only patients with severe ICD, which has a truly observable effect on their day-to-day life (e.g., substantial financial losses due to gambling). We deliberately decided to exclude patients with subclinical or borderline behavioral problems, because PD patients often manifest with a variety of behavioral problems, but only problems that differ from premorbid level of everyday functioning should be considered as a disorder. Hence, the inclusion of borderline patients would affect the validity of the study.

Secondly there is heterogeneity in our patient sample—we included patients with various manifestations of ICD. Future research studies could separately analyze individual subtypes of PD-ICD. Another limitation is the age difference between the PD-ICD and PD non-ICD patients. This factor needs to be considered, and caution should be exercised in interpretation of group differences. Moreover, not all of our patients were able to successfully undergo MRI testing; therefore, the sample for MRI testing was smaller than the sample for behavioral testing. However, the statistical comparison did not find any differences in self-reported impulsivity and behavioral performance between the subgroup of patients who underwent the MRI assessment and the group of patients without MRI testing. The last factor that may have influenced the results was the presence of some patients with a history of ICD. It is possible that previous personal experience could influence the behavior of these patients during the testing.

## Conclusion

The discrepancy between the presence of florid ICD signs, both in clinical follow-up and in self-reported impulsivity scales, and the comparable or even better performance of PD-ICD patients in rather well-established behavioral tasks is rather intriguing. This stalemate is further accentuated by significant structural differences in various neural nodes related to inhibitory control and reward processing in PD-ICD patients. Be it the lack of sensitivity in specific, limited ICD subtypes, excessive caution of ICD patients due to previous negative experience rendering simplistic tasks insufficient, or other, as of now unknown aspects, the issue of impulsivity in PD definitely warrants further research, ultimately to allow proactive prevention of this debilitating complication of PD.

## Data Availability Statement

The datasets generated for this study are available on request to the corresponding author.

## Ethics Statement

The studies involving human participants were reviewed and approved by Etická komise Masarykovy univerzity. The patients/participants provided their written informed consent to participate in this study.

## Author Contributions

PH, PF, PL, MS, MBal, MBar, and TK participated in designing the project and defining the aims and hypotheses. PF, RŠ, MBal, and MBar were responsible for patient recruitment and neurological examinations. PH and PL performed neuropsychological testing. PH and PF performed the data analysis and wrote the manuscript draft. All co-authors provided their comments to the manuscript draft.

### Conflict of Interest

The authors declare that the research was conducted in the absence of any commercial or financial relationships that could be construed as a potential conflict of interest.
